# Supplementary material for: Arabidopsis CaLB1 undergoes phase separation with the ESCRT protein ALIX and modulates autophagosome maturation
Source: Nat Commun. 2024 Jun 19;15:5188. doi: 10.1038/s41467-024-49485-6 (PMC11187125; doi:10.1038/s41467-024-49485-6)
Supplement: Supplementary file 1 — Supplementary Information [file 41467_2024_49485_MOESM1_ESM.pdf]

Supplementary Information

Arabidopsis CaLB1 undergoes phase separation with the ESCRT protein ALIX and modulates autophagosome maturation

Author list

Niccolò Mosesso[1], Niharika Savant Lerner[1,2], Tobias Bläske[1], Felix Groh[1], Shane Maguire[2,3], Marie Laura Niedermeier[2,4], Eliane Landwehr[2,5], Karin Vogel[1], Konstanze Meergans[1], Marie-Kristin Nagel[1], Malte Drescher[2,5], Florian Stengel[2,4], Karin Hauser[2,3], and Erika Isono[1,2,6 *]

Affiliations

[1]Plant Physiology and Biochemistry, Department of Biology, University of Konstanz, Universitätsstraße 10, 78457 Konstanz, Germany

[2]Konstanz Research School Chemical Biology, University of Konstanz, Universitätsstraße 10, 78457 Konstanz, Germany

[3]Biophysical Chemistry, Department of Chemistry, University of Konstanz, Universitätsstraße 10, 78457 Konstanz, Germany

[4]Biochemistry and Mass Spectrometry, Department of Biology, University of Konstanz, Universitätsstraße 10, 78457 Konstanz, Germany

[5]Spectroscopy of Complex Systems, Department of Chemistry, University of Konstanz, Universitätsstraße 10, 78457 Konstanz, Germany

[6]Division of Molecular Cell Biology, National Institute for Basic Biology, Nishigonaka 38, Myodaiji, Okazaki 444-8585 Aichi, Japan

*e-mail: erika.isono@uni-konstanz.de

Supplementary Table 1: Primers used in this study

Primer name	*Sequence (5‘ – 3‘)*
EI491 ATG8a qRT fw	TAACCCTCTCGAGGCAAGG
EI492 ATG8a qRT rv	TGTCAGGAACATCACTTTGTCC
EI493 ATG8b qRT fw	TTCAAGCTTTCTAATCCTCTGGA
EI494 ATG8b qRT rv	TCCAGCTTTTTCCACAATCAC
EI495 ATG8c qRT fw	TTTCAAGTTGGAACACCCACTA
EI496 ATG8c qRT rv	AGCTCTCTACGATCACTGGAA
EI497 ATG8d qRT fw	CGCTATTTCAGTCCCTTGGA
EI498 ATG8d qRT rv	TTCTCGGCTCTTTCGACAAT
EI458 ATG8e qRT fw	ACCCTGATCGAATTCCTGTG
EI459 ATG8e qRT rv	TTAGGTCTGATGGCACAAGGT
EI460 ATG8f qRT fw	TCCTGATAGGATTCCGGTGA
EI461 ATG8f qRT rv	AAACTGCCCCACAGTCAGAT
EI499 ATG8g qRT fw	CCCAAACATTGACAAGAAGAAA
EI500 ATG8g qRT rv	CATACACAAACTGGCCTACCG
EI501 ATG8h qRT fw	GGGGATTGTTGTCAAGTCTTTC
EI502 ATG8h qRT rv	TGCGTTTGAATATTTCTCAATGAT
EI509 ATG8i qRT fw	CGCTAAGTACCCTACTCGGATTC
EI510 ATG8i qRT rv	TCTCTTGGAACCAGAAACTTCTTT
FG79 VPS2.1 fw [Seq]	TATATAAAAATAATCTCGATTTTTCAACTCCGATCAAAGGTT
FG87 3xFLAG-RFP C fw	ATATGGTCTCACACCGTTCTCGACTACAAAGACCATGACGGTG
FG88 3xFLAGFP fw [Ovrlp]	CGATTACAAGGATGACGATGACAAGGTGTTGCTTATGGTGAGCAAGGGCGA
FG89 3xFLAGFP rv [Ovrlp]	TCGCCCTTGCTCACCATAAGCAACACCTTGTCATCGTCATCCTTGTAATCG
FG90 3xFLAGFP (D) rv [GG]	ATATGGTCTCTCCTTTTACTTGTACAGCTCGTCCATGC
FG105 3xFLAG-RFP B fw	CGATTACAAGGATGACGATGACAAGGTGTTGCTTATGGCCTCCTCCGAGG
FG106 3xFLAG-RFP C rv	CCTCGGAGGAGGCCATAAGCAACACCTTGTCATCGTCATCCTTGTAATCG
FG107 3xFLAG-RFP D rv	ATATGGTCTCTCCTTTTAGGCGCCGGTGGAGT
FG154 VPS2.1 Term RV rv [Geno]	CATAACCGAATTGAGAGGTGC
FG155 VPS2.1 rv [Geno]	CATTTTTCTAAGGTTATCCAACCTTGC
FG156 VPS2.1 fw [Geno]	ACTCTGAAATCTACACAAGCGA
GP16 T7 term	CTAGTTATTGCTCAGCGGT
GP17 T7 fw	TAATACGACTCACTATAGGG
GP22 ACT8 fw	TGAGACCTTTAATTCTCCAGCTATG
GP23 ACT8 rv	CCAGAGTCCAACACAATACC
GP41 eGFP rv	CTTGTGGCCGTTTACGTCG
KK40 ALIX BamHI fw	AAGGGGATCCTTGGCTTCTTCTTCGCTCT
KK41 ALIX SalI rv	AAGGGTCGACTCATTGCCTGTAGTATCCTCC
KK89 ∆BRO1 BamHI fw	AAGGGGATCCGAGAGATTACAGCAAGCT AGC
KK156 CaLB NdeI fw	AAGGCATATGTTGTCGATGATGGCG
KK157 CaLB BamHI rv	AAGGGGATCCCTAGTATGGAGGAGGTGGGTAT
MN439 A fw U6-26 Promotor	ATATGGTCTCAGCGGCGACTTGCCTTCCGCA
MN440 B rv U6-26 Terminator	ATATGGTCTCTCAGATATTGGTTTATCTCATCGGAACTG
MN443 C fw p35S-Cas9-NOS-SK	ATATGGTCTCACACCAAGCTTGCATGCCTGC
MN444 D rv p35S	ATATGGTCTCTCCTTCGTGTTCTCTCCAAATGAAATG
MN445 D fw CAS9	ATATGGTCTCAAAGGAACAATGGACTATAAGGACCACGAC
MN446 E rv CAS9	ATATGGTCTCTGATTTCACTTCTTCTTCTTCGCCT
MN447 E fw tNOS	ATATGGTCTCAAATCGAGCTCGAATTTCCCCGAT
MN448 G rv tNOS Cas9-NOS-SK	ATATGGTCTCTGACACCGATCTAGTAACATAGATGACAC
MN522 A fw proVPS2.1	ATATGGTCTCAGCGGGACAGAAAATATGATTTTACGGTTTT
MN523 B rv proVPS2.1	ATATGGTCTCTCAGAGATTCAATACCGAAAAAGGTGAA
MN528 B fw VPS2.1	ATATGGTCTCATCTGAACAATGATGAATTCAATCTTCGGAAAG
MN529 C rv VPS2.1	ATATGGTCTCTGGTGCCCATTTTTCTAAGGTTATCCAACCTT
MN572 Calbmut U6-26 rv	CGATAAGCGGACCTTGAATCAATCACTACTTCGACTCTAGC
MN573 Calbmut gRNA fw	GATTCAAGGTCCGCTTATCGGTTTTAGAGCTAGAAATAGCAAGTT
NM27 CaLB (No Stop) HindIII R	AAGGAAGCTTGTATGGAGGAGGTGGG
NM31 CaLB SALK_083243 fw	ACCAAGATGATTGCAACAG
NM32 CaLB SALK_083243 rw	CGTTCTTTCCACCATCTG

NM46 BsaI CaLB CRISPR_1 fw	ATTGATACAAGGCCAGATTCTCG
NM47 BsaI CaLB CRISPR_1 rv	AAACCGAGAATCTGGCCTTGTAT
NM78 pCaLB (1007 bp) TOPO fw	CACCGGTACAAGATATTCAGTTTAAGGG
NM80 gCaLB pFAST new TOPO rv	GGTATGGAGGAGGTGGGTATTGTC
NM89 CaLB GG A BsaI fw	ATATGGTCTCAGCGGGGTACAAGATATTCAGTTTAAGGG
NM90 CaLB GG B BsaI rv	ATATGGTCTCAGAATTTGGAAGAAAACAATCG
NM91 ProCaLB Mut* fw	TTATTTTGGACTCCGTTCTTC
NM92 ProCaLB Mut* rv	GAAGAACGGAGTCCAAAATAA
NM93 CaLB GG B BsaI fw	ATATGGTCTCAATTCATGTCGATGATGGCGG
NM94 CaLB GG C BsaI rv	ATATGGTCTCAGGTGGTATGGAGGAGGTGGGTATTGTC
NM99 BastaR GG D BsaI fw	ATATGGTCTCAAAGGTCTAGTAACATAGATGACACCGC
NM100 BastaR GG G BsaI rv	ATATGGTCTCAGACAGATCATGAGCGGAGAATTAAG
NM112 CaLB C2 BamHI rv	AAGGGGATCCTCACCCGTAATTATGTTTCTTTGCTC
NM113 CaLB PRD NdeI fw	AAGGCATATGGGGTCTGCGCCATCAGC
NM121 CaLB D32A Mut* fw	TTTCAAGACAAGCTCCATACGTCGTC
NM122 CaLB D32A Mut* rv	GACGACGTATGGAGCTTGTCTTGAAA
NM127 HsALIX CDS BamHI fw	AAGGGGATCCATGGCGACATTCATCTCG
NM128 HsALIX CDS EcoRI rv	AAGGGAATTCTTACTGCTGTGGATAGTAAGAC
NM130 CaLB NdeI fw	AAGGCATATGTCGATGATGGCGGG
NM132 CaLB aa 1-177 EcoRI rv	AAGGGAATTCTCATTGTTGTACTTGTGGGTATAGAG
NM133 CaLB aa 1-220 EcoRI rv	AAGGGAATTCTCAAGCTGAGGATGGCGGAG
NM149 CaLB(C2) HindIII rv	AAGGAAGCTTCCCGTAATTATGTTTCTTTGCTC
NM150 CaLB(1-177) HindIII R	AAGGAAGCTTTTGTTGTACTTGTGGGTATAGAG
NM157 gCaLB Intron 4 Mut* fw	CTTAAAACTGGACTCTTGAGGTA
NM158 gCaLB Intron 4 Mut* rv	TACCTCAAGAGTCCAGTTTTAAG 5'
NM159 mRFP+T35S GG C BsaI fw	ATATGGTCTCACACCTGGGACGTCCGCGGAGA
NM160 mRFP+T35S GG D BsaI rv	ATATGGTCTCACCTTAGGTCACTGGATTTTGGTTTTAGG
NM163 ScALIX BamHI fw	AAGGGGATCCATGAAACCTTACTTATTTGACCTA
NM164 ScALIX EcoRI rv	AAGGGAATTCCTAACTGCTGTATTTGGAGTAC
NM188 CaLB(CO) NdeI fw	AAGGCATATGAGTATGATGGCAGGGATTC
NM189 CaLB(CO) HindIII rv	AAGGAAGCTTATACGGAGGTGGCGGGTATTG
NM190 ALIX(CO) BamHI fw	AAGGGGATCCATGGCGAGTTCGTCCCTTAG
NM191 ALIX(CO) EcoRI rv	AAGGGAATTCTCATTGCCGATAGTAACCACCAC
NM196 ALIX GG C BsaI	ATATGGTCTCACACCATGGCTTCTTCTTCGCTCTCTAATC
NM237 CaLB(CO) SalI rv	AAGGGTCGACATACGGAGGTGGCGGGTATTG
NM271 CaLB C49S Mut* fw	ACCAGAACCAGCACAGATG
NM272 CaLB C49S Mut* rv	CATCTGTGCTGGTTCTGGT
NM273 CaLB C107S Mut* fw	TACGACGACAGTACCTGGAC
NM274 CaLB C107S Mut* rv	GTCCAGGTACTGTCGTCGTA
NM295 ATG8a attB1 fw	GGGGACAAGTTTGTACAAAAAAGCAGGCTTCATGGCTAAGAGTTCCTTCAAGATC
NM296 ATG8a attB2 rv	GGGGACCACTTTGTACAAGAAAGCTGGGTTCAAGCAACGGTAAGAGATCC
NM297 ATG8e attB1 fw	GGGGACAAGTTTGTACAAAAAAGCAGGCTTCATGAATAAAGGAAGCATCTTTAAG A
NM298 ATG8e attB2 rv	GGGGACCACTTTGTACAAGAAAGCTGGGTTTAGATTGAAGAAGCACCGAATG
NM299 ATG8i attB1 fw	GGGGACAAGTTTGTACAAAAAAGCAGGCTTCATGAAATCGTTCAAGGAACAATAC
NM300 ATG8i attB2 rv	GGGGACCACTTTGTACAAGAAAGCTGGGTTCAACCAAAGGTTTTCTCACTG
NM342 CaLB(C2)[N55C] Mut* fw	GGTGGAAAGTGCGCTGTTTTC
NM343 CaLB(C2)[N55C] Mut* rv	GAAAACAGCGCACTTTCCACC
NM350 CaLB(C2)[S29C] Mut* fw	CGGAATGGTTTTGTAGACAAGATCC
NM351 CaLB(C2)[S29C] Mut* rw	GGATCTTGTCTACAAAACCATTCCG
NM354 CaLB(C2)[L83C] Mut* fw	GAATAGCAACACTTGCTCCACTGATG
NM355 CaLB(C2)[L83C] Mut* rv	CATCAGTGGAGCAAGTGTTGCTATTC
NL1 ATG8f F	GGTCTCACACCATGGCAAAAAGCTCGTTCAAG
NL2 ATG8f R	GGTCTCACCTTTTATGGAGATCCAAATCCAAATGTGC
NL 47 ATG8a rv	TATAGTCGACTCATCCAAAAGTGTTCTCTCCA
NL 49 ATG8f rv	TATAGTCGACTCATCCAAATGTGTTTTCTCCGCTGTA
TB99 ALIX-PRD fw	TATCATATGAGCTCCGGCCCATACCCATC
TB100 ALIX-PRD rv	ATAGGATCCTTCATTGCCTGTAGTATCCTCC –
TB101 NdeI fw	ATACATATGATGTCGATGATGGCGGGTAT –

TB102 BamHI rv	TATGGATCCCCTAGTATGGAGGAGGTGGGT
TB112_GG_p35Spro_f	ATATGGTCTCAGCGGAGATTAGCCTTTTCAATTTCAG
TB113_GG_p35Spro_r	ATATGGTCTCTCAGACGTGTTCTCTCCAAATGAAATG
TB141 GG NOSt_fw	ATATGGTCTCAAAGGATCGTTCAAACATTTGGCAATAA
TB142 GG NOSt_rv	ATATGGTCTCTGATTTCTAGTAACATAGATGACACCG
TB161 ATG8a-NdeI fw	TATACATATGATCTTTGCTTGCTTGAAAT
TB169 ATG8f-SalI rv	TATACATATGGCAAAAAGCTCGTTCAAGC
TB175 ATG8i NdeI fw	TATACATATGAAATCGTTCAAGGAACAATAC
TB176 ATG8i SalI rv	TATAGTCGACTCAACCAAAGGTTTTCTCACTGC
TB179 GG 3xFlagC fw	ATATGGTCTCACACCGACTACAAAGACCATGACGGTG
TB180 GG 3xFlagC rv	ATATGGTCTCTCCTTTCACTTGTCATCGTCATCCTTG
TB245 CaLB AIM1 fw	CAAGATCCAGCCGTCGTCCTTG
TB246 CaLB AIM1 rv	CAAGGACGACGGCTGGATCTTG
TB247 CaLB AIM2 fw	GAGAAGTTTATCGCCACTTTGATTG
TB248 CaLB AIM2 rv	CAATCAAAGTGGCGATAAACTTCTC
TB249 CaLB AIM3 fw	GGACCATCTCTAGCCCCACAAG
TB250 CaLB AIM3 rv	CTTGTGGGGCTAGAGATGGTCC
TB251 CaLB AIM4 fw	CCTCGGGCGCCCCTCCGATC
TB252 CaLB AIM4 rv	GATCGGAGGGGCGCCCGAGG
TB270 GG NOSt fw	ATATGGTCTCATGAGTCTAGTAACATAGATGACACC
TB271 GG pOLE1 rv	ATATGGTCTCTGACAGTATGTAGGTATAGTAACATG
TB273 Mut RFP fw	GCTCGACGTACGTCTCTTTGTCG
TB274 Mut RFP rv	CGACAAAGAGACGTACGTCGAGC
TB275 Mut OLE fw	GTCACGGTCACGGCCCATCATCGGGTACTGATCTC
TB276 Mut OLE rv	GAGATCAGTACCCGATGATGGGCCGTGACCGTGAC
TB277 Mut pOLE fw	GATTCGGGACTCGTCCTCAAG
TB278 Mut pOLE rv	CTTGAGGACGAGTCCCGAATC
TB306 pGEX Mut fw	CACAGGAAACAGAATTCATGTCCC
TB306 pGEX Mut rv	GGGACATGAATTCTGTTTCCTGTG
TB307 pGEX-MCS fw	TATAGAATTCATATGCAAAGCTTCAGTCGACTGGGATCCACTGGAGAGACCTATA
TB308 pGEX-MCS rv	TATAGGTCTCTCCAGTGGATCCCAGTCGACTGAAGCTTTGCATATGAATTCTATA
TB309 pGEX-PreSc fw	ATATGGTCTCACTGGAAGTTCTGTTCCAGGGGATGTAGAGACCATAT
TB310 pGEX-PreSc rv	ATATGGTCTCTACATCCCCTGGAACAGAACTTCCAGTGAGACCATAT
TB311 pGEX-GST fw	ATATGGTCTCTATGTCCCCTATACTAGGTTATTGG
TB312 pGEX-GST rv	ATATCTCGAGTCAATCCGATTTTGGAGGATGGTCG
TB450 ALIX-GG-fw	ATATGGTCTCTCCTTTCATTGCCTGTAGTATCCTCC
TB468 CaLB qRT fw	CATATTACCCACAAG GTCCATATCC
TB469 GFP qRT rv	CTTGCTCACCATCCGC
TB472 CaLB qRT rv	CACCATACTCAAGGACGACG
TB506 ALIX-qRT1 fw	CACTCTCCAAGATGCAATCACG
TB507 ALIX-qRT1 rv	CCAGACATCTGTCGTTGCAC
TB575 CaLB qRT fw	TCGAGGTCACTGTTGTTGG
O8474 (GabiKat LB)	ATAATAACGCTGCGGACATCTACATTTT

Supplementary Table 2: Vectors and plasmids used in this study

Plasmid name	*Description*	*Backbone*	*Source*
pDONR207	Gateway entry vector		Thermo Fisher Scientific
pENTR/D-TOPO	Gateway entry vector		Thermo Fisher Scientific
pET21a(+)	N-term T7, C-term 6xHis, Amp^R		Merck Millipore
pET28a(+)	N-term 6xHis-thrombin site-T7 tag, C-term 6xHis, Kan^R		Merck Millipore
pGWB404	Gateway destination vector, C-Term sGFP, Sp^R, Kan^R		[1]
pGWB411	Gateway destination vector, 35S pro, C-term FLAG, Sp^R, Kan^R		[1]
pGWB614	Gateway destination vector, 35S pro, C-term 3xHA, Sp^R, $BASTA^R$		[1]
pFAST-R05	Gateway destination vector, C-term GFP, OLE1-TagRFP, Sp^R, Kan^R		[2]
pFAST-R07	Gateway destination vector, 35S pro, C-term GFP, OLE1-TagRFP, Sp^R, Hyg^R		[2]
pHEE401E	Golden Gate, CRISPR/Cas9		[3]
pGADT7	YTH HA-GAD, Amp^R		Takara Bio
pGBKT7	YTH myc-GBD, Kan^R		Takara Bio
pGEX-6P-1	N-term GST, Amp^R		Cytiva
pLIIA F1-2	GoldenGate (pBB10)		[4]
pMAL-p2T	MBP, Thrombin site, Amp^R		Keiji Tanaka lab
pMAL-p2p	MBP, PreScission site, Amp^R		Keiji Tanaka lab
pUBN-GFP-Dest	Gateway destination vector, N-term GFP		[5]
pUBN-mRFP-Dest	Gateway destination vector, N-term mRFP		[5]
pUBC-GFP-Dest	Gateway destination vector, C-term GFP		[5]
pUBC-mRFP-Dest	Gateway destination vector, C-term mRFP		[5]
pUC19	For subcloning, Amp^R		Takara Bio
pUC57	For subcloning, Amp^R		Thermo Fisher Scientific
pJET1.2	For subcloning, Amp^R		Thermo Fisher Scientific
pBB02	For Golden Gate assembly, $Gent^R$	pUC57	this study
pFG41	VPS2.1pro:VPS2.1-3xFLAG-GFP	pBB10	this study
pFG44	3xFLAG-sGFP	pJET1.2	this study
pFG45	3xFLAG-mRFP	pJET1.2	this study
pFG46	VPS2.1pro:VPS2.1-3xFLAG-mRFP	pBB10	this study
pKK28	MBP-VPS60.1(BRO1)	pMAL-p2T	[6]
pKK31	MBP-ALIX(BRO1)	pMAL-p2p	[6]
pKK32	MBP-ALIX(ΔBRO1)	pMAL-p2p	this study
pKK41	GBD-Myc-ALIX	pGBKT7 (NdeI/BamHI)	[6]
pKK48	GST-ALIX(CDS)	pGEX-6P-1	this study
pKK49	GBD-Myc-ALIX(BRO1)	pGBKT7 (NdeI/BamHI)	[6]
pKK50	GBD-Myc-ALIX(ΔBRO1)	pGBKT7 (NdeI/BamHI)	[6]
pKK51	GBD-Myc-ALIX(ΔC)	pGBKT7 (NdeI/BamHI)	[6]
pKK90	GAD-HA-CaLB	pGADT7 (NdeI/BamHI)	this study
pMN173	35Spro	pJET1.2	this study
pMN174	tNOS	pJET1.2	this study
pMN177	CAS9	pJET1.2	this study
pMN199	CRIPSR(CalB) Exon 1 U6-26	pJET1.2	this study
pMN200	noncrCalB Exon 1 U6-26	pJET1.2	this study
pMN201	CRISPR(CalB) Exon 1	pBB02	this study
pMN202	CRISPR (mutCalB) Exon 1	pBB02	this study
pNM7	CaLB-6xHis	pET21a(+) (NdeI/HindIII)	this study
pNM10	U6-26_{Pro}:sgRNA(CaLB_exon1)	pHEE401E (BsaI)	this study

pNM17	CaLB$_{Pro}$:gCaLB for pFAST-R07	pENTR/D-TOPO	this study
pNM22	CaLB$_{Pro}$:gCaLB-eGFP	pFAST-R07	this study
pNM36	CaLB(CDS w/o stop)	pUC19 (XbaI/HindIII)	this study
pNM39	GAD-HA-CaLB(C2)	pGADT7 (NdeI/BamHI,)	this study
pNM40	GAD-HA-CaLB(PRD)	pGADT7 (NdeI/BamHI,)	this study
pNM41	GAD-HA-CaLB(aa1-177)	pGADT7 (NdeI/BamHI,)	this study
pNM42	GAD-HA-CaLB(aa1-220)	pGADT7 (NdeI/BamHI,)	this study
pNM45	CaLB(C2)-6xHis	pET21a (+) (NdeI/HindIII)	this study
pNM46	CaLB(1-177)-6xHis	pET21a (+) (NdeI/HindIII)	this study
PNM48	GST-hALIX	pGEX-6P1 (BamHI/EcoRI,)	this study
pNM52	CaLB$_{Pro}$:gCaLB-mRFP	pLIIA F1-2	this study
pNM54	GST-ScBro1	pGEX-6P1 (BamHI/EcoRI,)	this study
pNM64	CaLB (codon optimized) w/o stop	pEX-K168 (Eurofins)	this study
pNM65	ALIX (codon optimized) w/o stop	pEX-K248 (Eurofins)	this study
pNM66	CaLB (codon optimized)-6xHis	pET21a (+) (NdeI/HindIII,)	this study
pNM67	GST-ALIX (codon optimized)	pGEX-6P-1 (BamHI/EcoRI,)	this study
pNM68	CaLB (CDS, codon optimized)-GST	pTB111	this study
pNM93	CaLB(1-177)[C49S]-6xHis	pET21a (+) (NdeI/HindIII)	this study
pNM97	CaLB(1-177)[C49S/C107S]-6xHis	pET21a (+) (NdeI/HindIII)	this study
pNM101	CaLB[D32A](C2)-6xHis	pET21a (+) (NdeI/HindIII)	this study
pNM103	ATG8a (with stop)	pDONR207	this study
pNM104	ATG8e (with stop)	pDONR207	this study
pNM105	ATG8i (with stop)	pDONR207	this study
pNM106	UBQ10$_{Pro}$:mRFP-ATG8a	pUBN-mRFP-Dest	this study
pNM107	UBQ10$_{Pro}$:GFP-ATG8a	pUBN-GFP-Dest	this study
pNM108	UBQ10 $_{Pro}$:mRFP-ATG8e	pUBN-mRFP-Dest	this study
pNM110	UBQ10 $_{Pro}$:mRFP-ATG8i	pUBN-mRFP-Dest	this study
pNM117	CaLB $_{Pro}$:gCaLB[F64A](w/o stop)-eGFP	pFAST-R07 (XhoI/BstXI)	this study
pNM125	CaLB(1-177)[C49S/C107S/S29C]-6xHis	pET21a (+) (NdeI/HindIII)	this study
pNM127	CaLB(1-177)[C49S/C107S/L83C]-6xHis	pET21a (+) (NdeI/HindIII)	this study
pNM129	CaLB(1-177)[C49S/C107S/N55C]-6xHis	pET21a (+) (NdeI/HindIII)	this study
pTB32	GBD-Myc-ALIX(PRD)	pGBKT7 (NdeI/BamHI)	this study
pTB63	NOS terminator	pJET2.1	this study
pTB66	3XFLAG	pJET2.1	this study
pTB70	GBD-Myc-ATG8i	pGBKT7 (NdeI/SalI)	this study
pTB85	OLE1pro:OLE1-TagRFP:NOSter	pJET2.1	this study
pTB102	pBB02 CmR-ccdB	pBB02	[7]
pTB103	EcoRI site upstream of GST	pGEX-6P-1	this study
pTB111	MCS-PreScission site-GST	pGEX-6P-1	this study
pTB204	sGFP-ALIX	pBB02	this study
pNL1	mRFP-ATG8f	pBB02	this study
pNL7	6xHis-T7tag-ATG8	pET28a(+) (NdeI/SalI)	this study
pNL8	6xHis-T7tag-ATG8f	pET28a(+) (NdeI/SalI)	this study
pLK3	6xHis-T7tag-ATG8i	pET28a(+) (NdeI/SalI)	this study
pYP1	GAD-HA-CaLB[Y34A]	pGADT7 (NdeI/BamHI)	this study
pYP2	GAD-HA-CaLB[F64A]	pGADT7 (NdeI/BamHI)	this study
pYP3	GAD-HA-CaLB[Y172A]	pGADT7 (NdeI/BamHI)	this study
pYP4	GAD-HA-CaLB[Y205A]	pGADT7 (NdeI/BamHI)	this study
pJW3	CaLB[D32A]	pUC19	this study
pJW6	CaLB[D32A]-6xHis	pET21a(+) (XbaI/HindIII)	this study

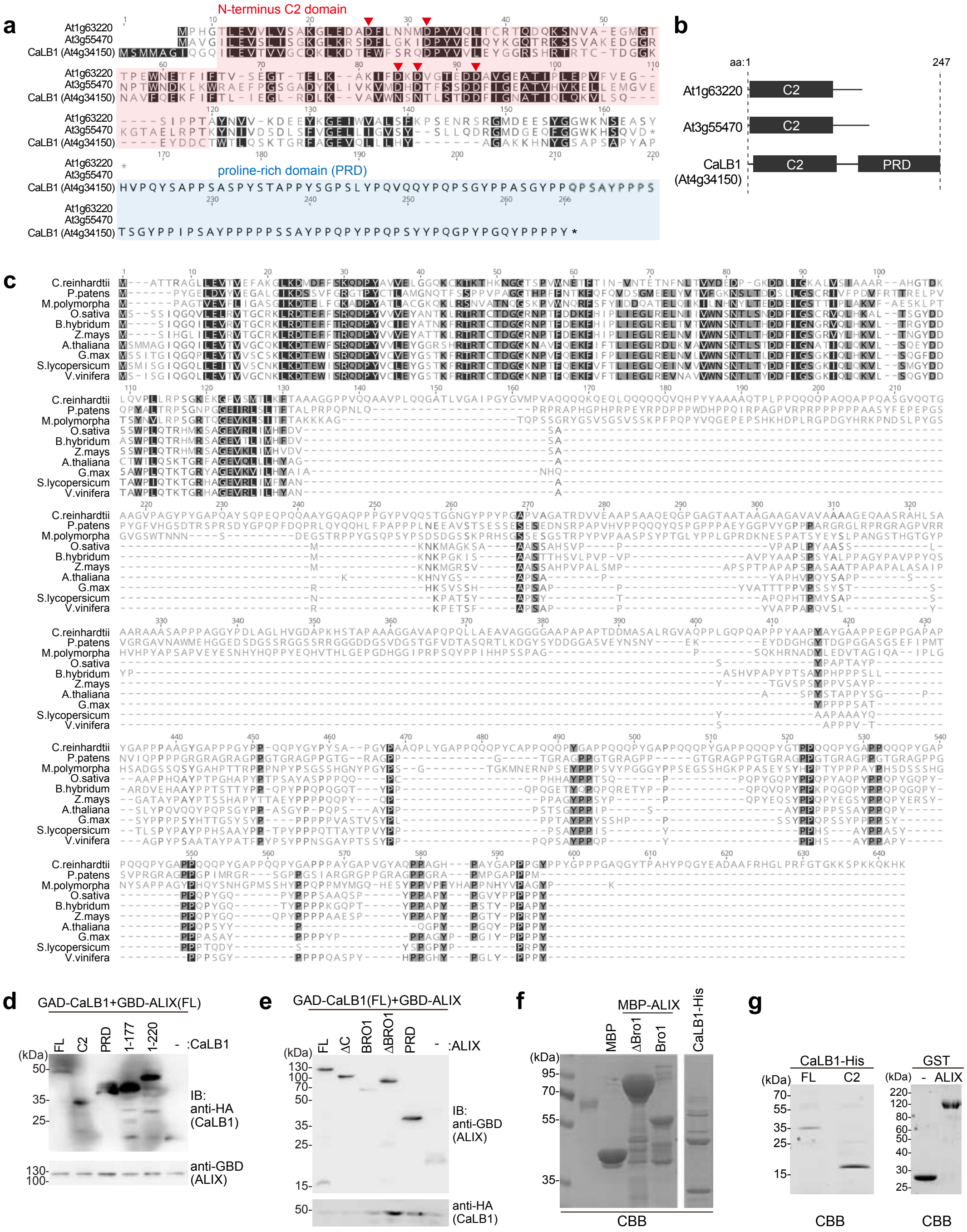

Supplementary Figure 1: CaLB1 is conserved in the green lineage and interacts with ALIX.

(a) Alignment of the amino acid sequences of CaLB1 (At4g34150), At1g63220, and At3g55470. Identical amino acids or amino acids with similar properties are highlighted with a dark background. Note that whereas CaLB1 has a C-terminus proline rich domain, the other two do not.

(b) Domain organization of CaLB1 (At4g34150), At1g63220, and At3g55470.

(c) Alignment of the amino acid sequences of CaLB homologs in other plant species. Identical amino acids or amino acids with similar properties are highlighted with a dark background. C. reinhardtii: *Chlamydomonas reinhardtii* Cre17.g723700 t1.1, P. patens: *Physcomitrella patens* Pp3c1_41500V3.1.p, M. polymorpha: Marchantia polymorpha Mapoly0021s0067.1.p, B. hybridum: Brachypodium hybridum Brahy.D01G0398400.1.p, O. sativa: *Oryza sativa* Os06g43190.1, Z. mays: *Zea mays*, Zm00001d046625_P001, A. thalina: *Arabidopsis thaliana* CaLB1 (At4g34150), G. max: *Glycine max* Glyma.01G244500.1.p, S. lycopersicum: *Solanum lycopersicum* Solyc01g107740.3.1, and V. vinifera: *Vitis vinifera* VIT_203s0180g00030.1. Sequence alignment was generated in Geneious Prime (vers. 2022.2.2 (restricted)) using the Geneious Alignment tool.

(d) Immunoblot for the yeast two-hybrid clones shown in Fig. 1d. Yeast total extracts were subjected to Immunoblot using an anti-HA antibody to detect GAD-fused CaLB1 variants and an anti-GBD antibody to detect GBD-fused ALIX. Source data are provided as a Source Data file.

(e) Immunoblot for the yeast two-hybrid clones shown in Fig. 1e. Yeast total extracts were subjected to Immunoblot using an anti-HA antibody to detect GAD-fused CaLB1 and an anti-GBD antibody to detect GBD-fused ALIX variants. Source data are provided as a Source Data file.

(f) Purification of recombinant CaLB1-6xHis and MBP-fused ALIX(ΔBro1) and ALIX(Bro1) used for the in vitro binding assay in 1f. Recombinant proteins were isolated from *E. coli*, resolved on an SDS-PAGE gel and stained with CBB. Source data are provided as a Source Data file.

(g) Purification of recombinant CaLB1-6xHis and CaLB1(C2)-6xHis, GST, and GST-ALIX used for the in vitro binding assay in 1g. Recombinant proteins were isolated from *E. coli*, resolved on an SDS-PAGE gel and stained with CBB. Source data are provided as a Source Data file.

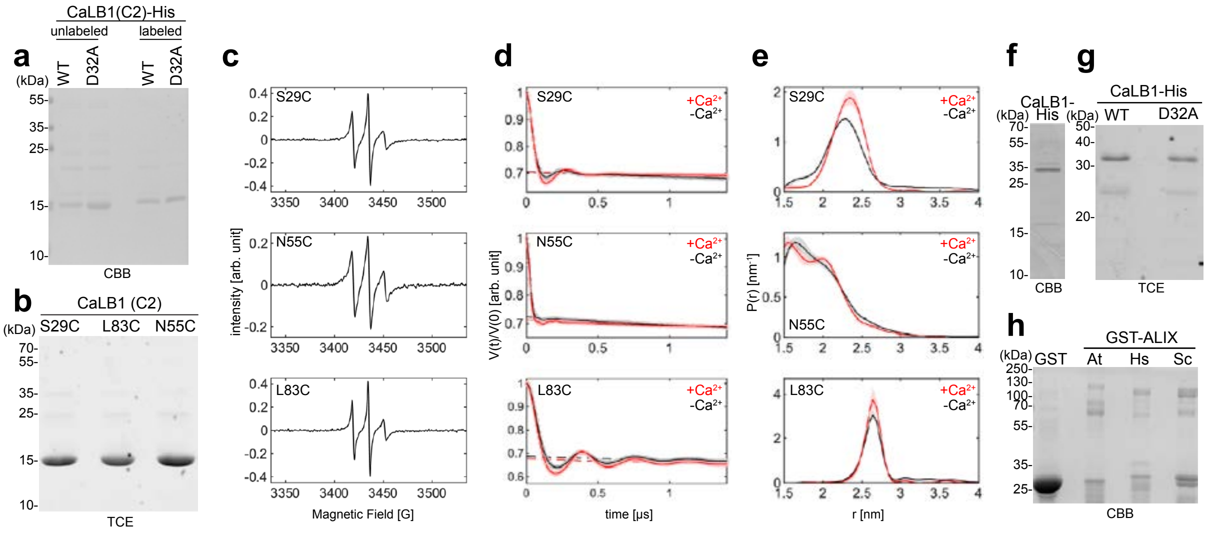

Supplementary Figure 2: CaLB binds Ca^{2+} ions.

(a) Purification and labelling of recombinant CaLB1 used for the MST analysis in Fig. 2b. Recombinant CaLB1(C2)-6xHis and CaLB1[C2(D32A)] were isolated from *E. coli* and labelled with the GREEN-maleimide fluorescent dye. Proteins were resolved on an SDS-PAGE and stained with CBB. Source data are provided as a Source Data file.

(b) Purification of recombinant CaLB1 used for the EPR spectroscopy shown in (c) to (e). Recombinant CaLB1[C2(S29C)]-6xHis, CaLB1[C2 (N55C)]-6xHis, and CaLB1[C2(L83C)]-6xHis were isolated from *E. coli* and resolved on an SDS-PAGE and protein bands were visualized using trichloroethanol (TCE). Source data are provided as a Source Data file.

(c) X-band EPR spectra at ambient temperature of CaLB1(C2) variants S29C, N55C, and L83C after site-directed spin labeling with S-(1-oxyl-2,2,5,5-tetramethyl-2,5-dihydro-1H-pyrrol-3-yl)methyl methanesulfonothioate (MTSL). Continuous wave spectra show the attachment of the spin label to the proteins.

(d)(e) Raw DEER data on spin-labelled CaLB1(C2) variants S29C, N55C, and L83C including fits (continuous lines) and intermolecular background (dashed lines) (d) as well as distance distributions including 95 % confidence interval (e). Measurements were performed either without calcium (black) or with 10 mM $CaCl_2$(red). arb. unit: arbitrary unit.

(f) Purification of recombinant His-tagged CaLB1 used for the lipid overlay assay shown in Fig. 2c. Recombinant CaLB1-6xHis was isolated from *E. coli* and resolved on an SDS-PAGE and stained with CBB. Source data are provided as a Source Data file.

(g) Purification of recombinant CaLB1 used for the ATR-IR spectroscopy shown in Fig 2d. Recombinant CaLB1-6xHis and CaLB(D32A)-6xHis were isolated from *E. coli* and resolved on an SDS-PAGE and protein bands were visualized using trichloroethanol (TCE). Source data are provided as a Source Data file.

(h) Purification of recombinant GST-ALIX and GST-ScBro1 used for the lipid overlay assay shown in Fig. 2e. Recombinant GST, GST-ALIX and GST-ScBro1 were isolated from *E. coli* and resolved on an SDS-PAGE and stained with TCE. Source data are provided as a Source Data file.

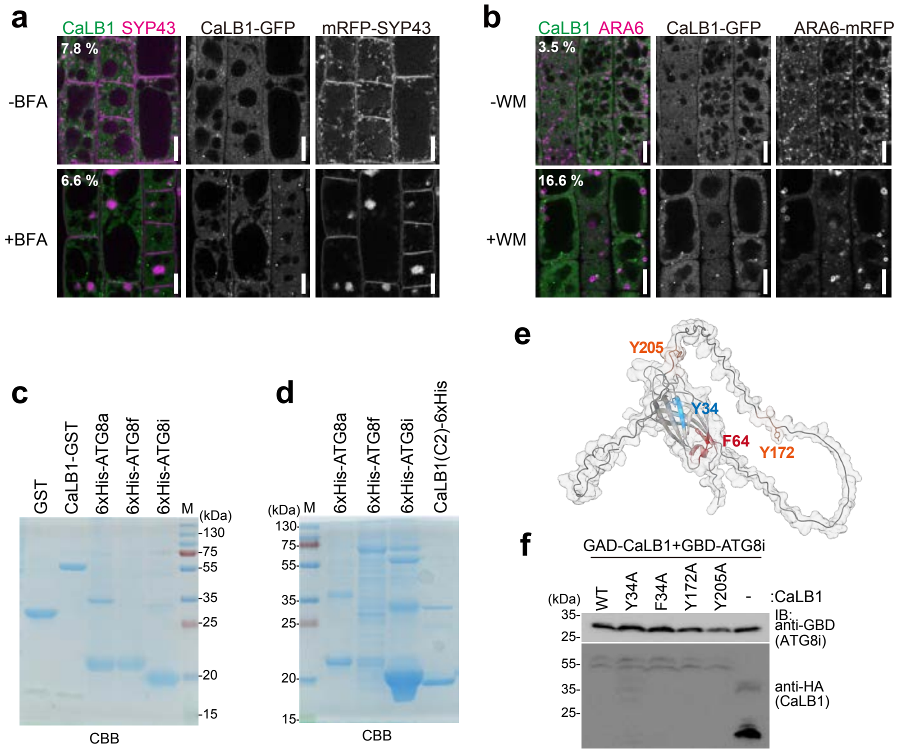

Supplementary Figure 3: CaLB interacts with ATG8.

(a) Colocalization of CaLB1-GFP and mRFP-SYP43. Prior to analysis, seedlings were treated with DMSO [-brefeldin A (BFA)] or with 50 µM of BFA (+BFA) for 60 minutes. 7.8% (n=565, 10 images) and 6.6% (n=2640, 38 images) of CaLB1-positive foci showed colocalization with mRFP-SYP43 under -BFA and +BFA conditions, respectively. Scale bars: 10 µm. The experiment was conducted twice and representative images are shown.

(b) Colocalization of CaLB1-GFP and ARA6-mRFP. Prior to analysis, seedlings were kept in 1/2 MS media with DMSO [-Wortmannin (WM)] or with 33 µM WM (+WM) for 90 minutes. 3.5% (n=941, 11 images) and 16.6% (n=241, 7 images) of CaLB1-positive foci showed colocalization with ARA6-mRFP under -WM and +WM conditions, respectively. Scale bars: 10 µm. The experiment was conducted twice and representative images are shown.

(c) Recombinant proteins used for the in vitro binding assays shown in Fig. 3a. Recombinant GST, CaLB-GST, 6xHis-ATG8a, -ATG8f, and -ATG8i were isolated from E. coli and resolved on an SDS-PAGE and protein bands were visualized by staining with CBB. Source data are provided as a Source Data file.

(d) Recombinant proteins used for the MST analysis shown in Fig. 3b. Recombinant CaLB1(C2)-6xHis and 6xHis-ATG8i were isolated from E. coli and resolved on an SDS-PAGE and protein bands were visualized by staining with CBB. Source data are provided as a Source Data file.

(e) AlphaFold model of CaLB1. The side chains of the mutated amino acids in Figure 3c are shown. Blue: Y34-VVL, red:F64-TLI, orange: Y172-PQV and Y205-PPI.

(f) Immunoblot for the yeast two-hybrid clones shown in Fig. 3c. Yeast total extracts were subjected to Immunoblot using an anti-HA antibody to detect GAD-fused CaLB1 variants and an anti-GBD antibody to detect GBD-fused ATG8i. Source data are provided as a Source Data file.

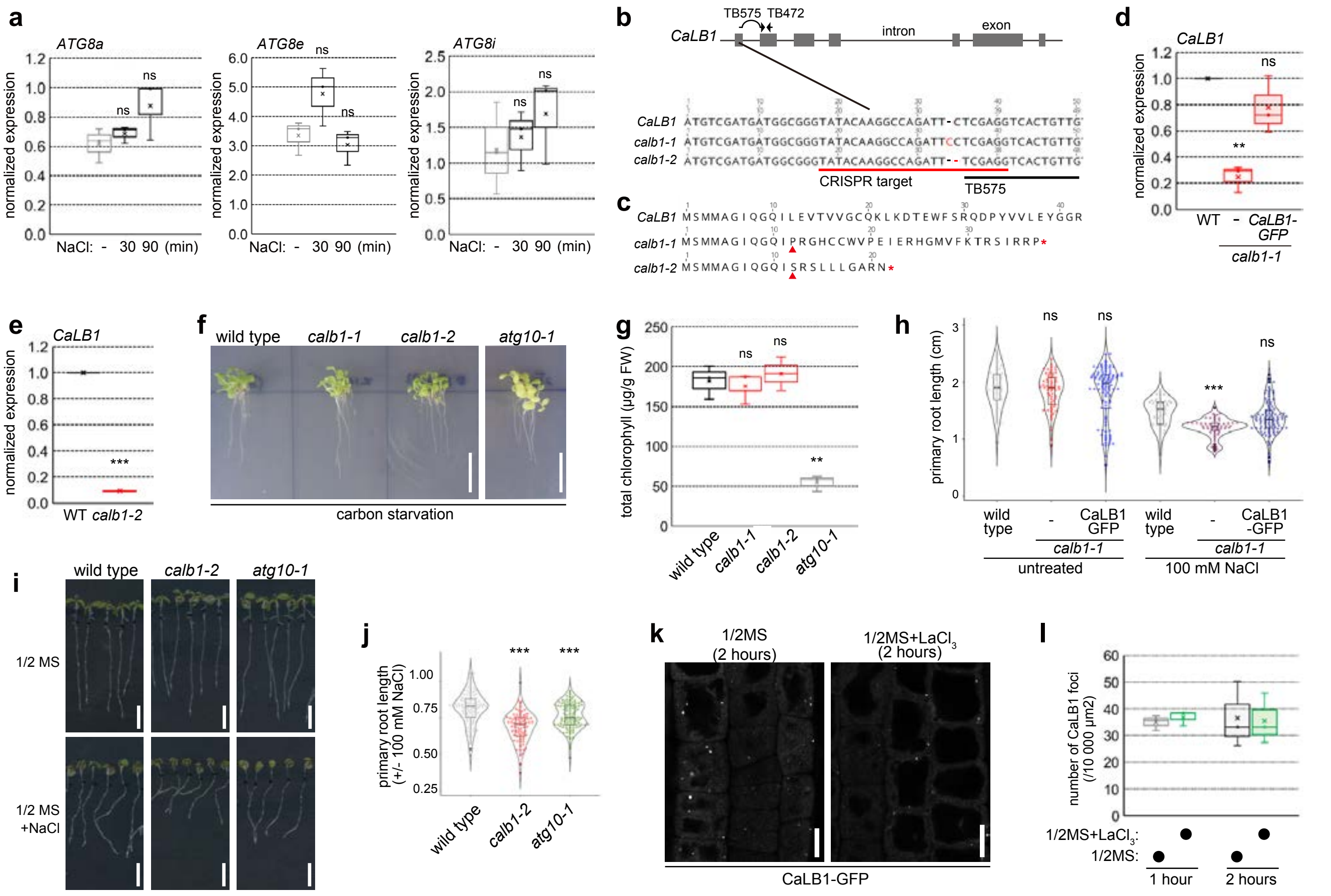

Supplementary Figure 4: *calb1* mutants are sensitive to salt treatment.

(a) Expression of *ATG8*s upon NaCl treatment normalized against *ACTIN8*. The experiment was conducted three times with technical quadruplicates each. Average expression for each experiment is shown. *ATG8a*: untreated/30 min p=0.416 (ns: not significant, p>0.5), untreated/90 min p=0.141 (ns), *ATG8e*: untreated/30 min p=0.116 (ns), untreated/90 min p=0.561 (ns), *ATG8i*: untreated/30 min p=0.717 (ns), untreated/90 min p=0.381 (ns).

(b) CRISPR target and indel events in *calb1-1* and *calb1-2*. TB575: primer for qRT-PCR used in (d).

(c) Protein sequence of *calb1-1* and *calb1-2*. Arrowhead: positions of the indel mutation, asterisk: Premature stop codon.

(d)(e) Expression of CaLB1 in *calb1-1* and *calb1-1* with *CaLB1pro:CaLB1-GFP* (d) or in *calb1-2* (e). qRT-PCR was performed three times with four technical replicates each. Expression was normalized against ACTIN8 and the wild-type value was set to 1. Wild type/*calb1-1* p= 0,00617 (**: 0.001<p<0.01), wild type/*calb1-1* with CaLB1-GFP p= 0.226 (ns: p>0.5). Wild type/*calb1-2* p= 1.12x10-5 (***: p<0.001).

(f) Wild-type, *calb1-1*, *calb1-2*, and *atg10-1* seedlings after 6 days of incubation in darkness. Scale bars: 1.5 cm. -C: carbon starvation.

(g) Chlorophyll content of seedlings in (f). Wild type/*calb1-1* p= 0.739 (ns, p>0.5), wild type/*calb1-2* p= 0.625 (ns, p>0.5), wild type/*atg10-1* p= 0.00308 (**, 0.1<p<0.5),. The experiment was conducted three times and all results are shown. FW: fresh weight.

(h) Primary root length of 7-day-old wild type (+/- NaCl, n=53 and n=49 seedlings, respectively), *calb1-1* (+/- NaCl, n=53 and n=65 seedlings, respectively), *calb1-1* expressing CaLB1-GFP (+/- NaCl, n=100 seedlings for both). -NaCl wild type/*calb1-1* p=0.347 (ns, p>0.5), wild type/*calb1-1* expressing *CaLB1-GFP* p=0.198 (ns, p>0.5), +100 mM NaCl wild type/calb1-1 p=2.52*10-8 (***: p<0.001), wild type/*calb1-1* expressing *CaLB1-GFP* p=0.0675 (ns, p>0.5). The experiment was conducted three times and one representative result is shown.

(i) 7-day-old wild type, *calb1-1*, and *atg10-1* with and without NaCl treatment. Scale bars: 5 mm.

(j) Primary root length of seedlings treated as in (i). Wild type (n=99 and 91 for +/- NaCl, respectively), *calb1-2* (n=99 and 91 for +/- NaCl, respectively), *atg10-1* (n=92 and 99 for +/- NaCl, respectively). Root length after NaCl treatment was divided by the average root length of the untreated one. Wild type/*calb1-2* p= 4.60x10-12 (***: p<0.001), wild type/*atg10-1* p=6.12x10-4 (***: p<0.001). The experiment was conducted twice and one result is shown.

(k) Effect of 150 µM Lanthanum(III) chloride ($LaCl_3$) on the localization of CaLB1-GFP. The experiment was conducted three times and representative images are shown. Scale bars: 10 µm.

(l) Quantification of the results in (k). 1h MS/$LaCl_3$ p= 0.446 (ns, p>0.5), 2h MS/$LaCl_3$ p=0.915 (ns, p>0.5). The experiment was conducted three times and the average of each experiment is shown.

(a, d, e, g, h, j, l) Box plot: center line, median; box limits, first and third quartiles; whiskers, 1.5x interquartile range; points, outliers. Two-tailed t-test, no equal variance. Source data are provided as a Source Data file.

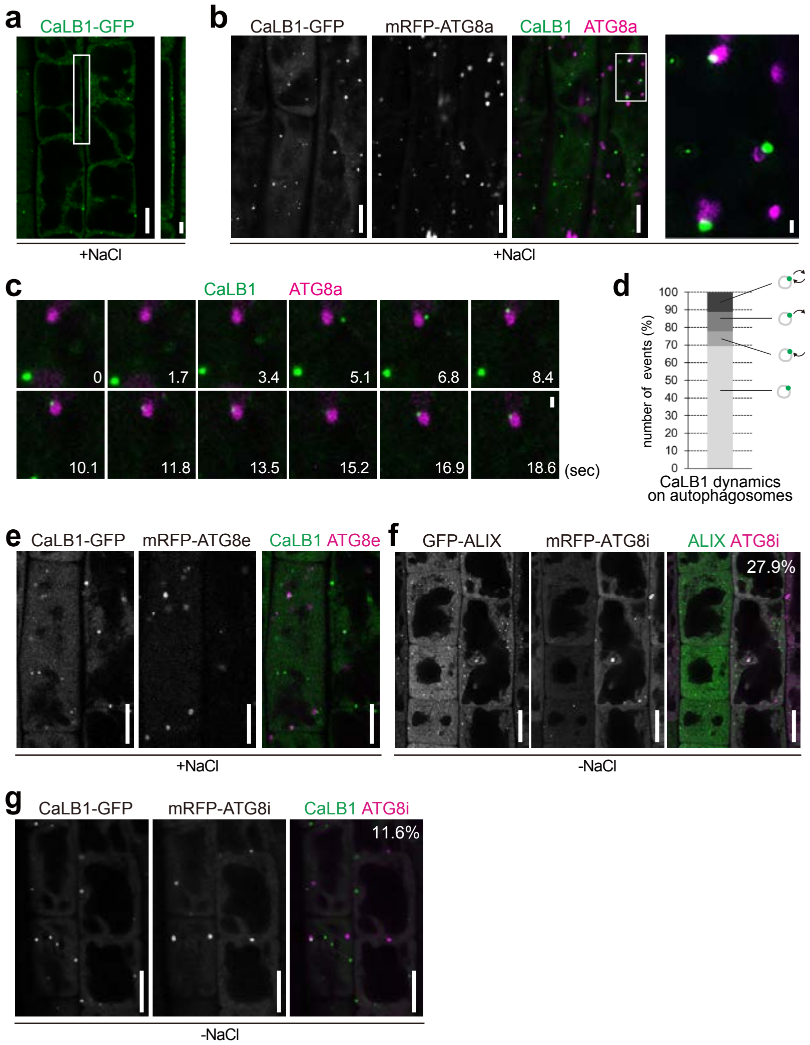

Supplementary Figure 5: CaLB1 and ALIX localize on ATG8-marked autophagosomes.

(a) Relocalization of CaLB1-GFP signals to plasma membrane. After 1.5 hours of 150 mM NaCl treatment of the CaLB1-GFP-expressing seedlings, occasional relocalization of CaLB1-GFP to me plasma membrane was observed. Rectangle: magnified region. Scale bars: 10 um, in the magnification: 2 µm.

(b) Localization of CaLB1-GFP and mRFP-ATG8a in root epidermis cells. 5-day-old seedlings expressing *CaLB1pro:CaLB1-GFP* and *UBQ10pro.mRFP-ATG8a* were grown in 1/2 MS media and treated with 150 mM NaCl for 2 hours before confocal imaging. Scale bars: 10 µm. Rectangle: magnified region. The experiment was conducted twice and one image sequence is shown. Scale bars in the magnification: 1 µm.

(c)(d) Analyses of the dynamics of CaLB1 localization on autophagosome was conducted on time-lapse images of 5-day-old seedling expressing *CaLB1pro:CaLB1-GFP* and *UBQ10pro mRFP-ATG8a* treated for 2 hours in 1/2 MS supplemented with 150 mM NaCl. Confocal images in (c) were taken at the indicated time points. Time-lapse images of 36 autophagosomes from 17 seedlings were quantified. Scale bars: 1 µm. Source data are provided as a Source Data file (d).

(e) Localization of CaLB1-GFP and mRFP-ATG8e in root epidermis cells. 5-day-old seedlings expressing *CaLB1pro:CaLB1-GFP* and *UBQ10pro:mRFP-ATG8e* were grown in 1/2 MS media and treated with 150 mM NaCl for 2 hours before confocal imaging of root epidermis cells. Scale bars: 10 µm.

(f) Localization of GFP-ALIX and mRFP-ATG8i in root epidermis cells. 5-day-old seedlings expressing *ALIXpro:GFP-ALIX* and *UBQ10pro:mRFP-ATG8i* were grown in 1/2 MS media and moved in liquid 1/2 MS media for 2 hours before confocal imaging of root epidermis cells. 27.9% of autophagosomes (n=408 from 17 seedlings) were also positive for GFP-ALIX signals. Scale bars: 10 µm.

(g) Localization of CaLB1-GFP and mRFP-ATG8i in root epidermis cells. 5-day-old seedlings expressing *CaLB1pro:CaLB1-GFP* and *UBQ10pro:mRFP-ATG8i* were grown in 1/2 MS media and moved in liquid 1/2 MS media for 2 hours before confocal imaging of root epidermis cells. 11.6% of autophagosomes (n=250 from 17 seedlings) were also positive for CaLB1-GFP signals. Scale bars: 10 µm.

(a, e, f, g) The experiments were conducted three times and representative images are shown.

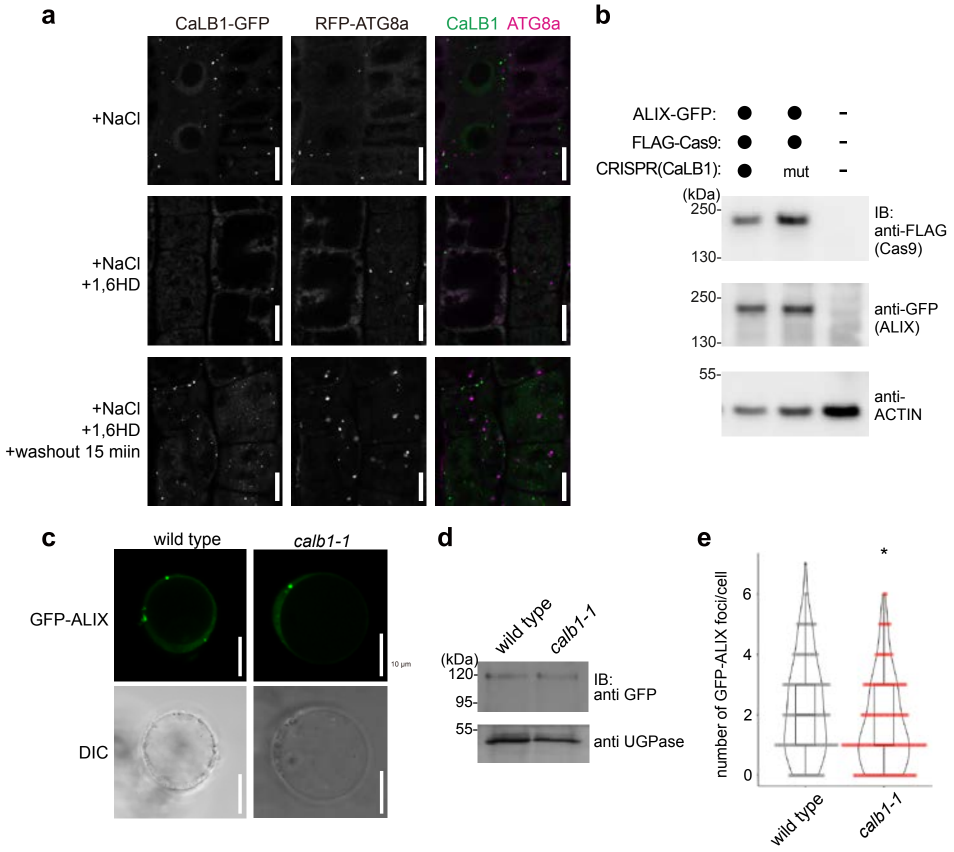

Supplementary Figure 6: CaLB1 and ALIX form molecular condensates in vivo.

(a) *CaLB1pro:CaLB1-GFP* and *UBQ10pro:RFP-ATG8i* expressing seedlings were treated for 2 hours with 150 mM NaCl or 150 mM NaCl followed by 1 minute incubation with 5% 1,6 hexanediol (1,6HD) as in Figure 6c. For the washout of 1,6HD, seedlings were rinsed twice and incubated for 15 minutes in liquid 1/2 MS media supplemented with 150 mM NaCl before imaging. Note that though fewer CaLB1-GFP foci are visible after 1,6HD treatment whereas after washout of 1,6HD the number of GFP-positive foci recovers. The experiment was conducted three times and representative images are shown. Scale bars: 10 µm.

(b) Immunoblot of total extracts from protoplasts analyzed in Fig. 6i and 6j. An anti-FLAG antibody and an anti-GFP antibody were used to detect the FLAG-tagged Cas9 and GFP-fused ALIX. An anti-ACTIN antibody was used for loading control. Source data are provided as a Source Data file.

(c) Representative images of Arabidopsis protoplasts. Protoplasts derived from wild-type and *calb1-1* seedling roots were transformed with *UBQ10pro:GFP-ALIX* and analyzed under a confocal microscope. The experiment was conducted four times and representative images are shown. Scale bars: 10 µm.

(d) Immunoblot of protoplasts. Total extracts were isolated from wild type and *calb1-1*-derived root cell protoplasts transformed with *UBQ10pro:GFP-ALIX*. An anti-GFP antibody was used to detect GFP-ALIX. An anti-UGPase antibody was used as loading control. Four replicates were analyzed and one representative result is shown. Source data are provided as a Source Data file.

(e) Violin plot of the number of GFP-ALIX-positive foci per protoplast transformed as in (c) [n=124 (wild type), n=138 (*calb1-1*)]. Center line, median; box limits, first and third quartiles; whiskers, 1.5x interquartile range. p-values for the number of ALIX foci in wild type and calb1-1 protoplasts: p=0.0102 (*: 0.01<p<0.05), two-tailed t-test, no equal variance. Source data are provided as a Source Data file.

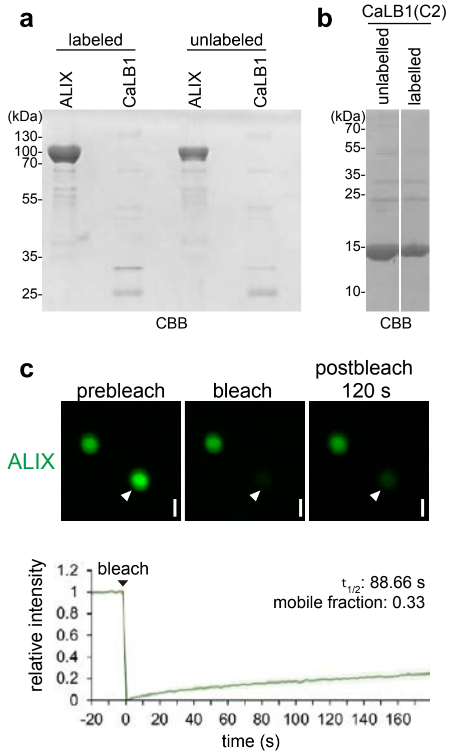

Supplementary Figure 7: CaLB and ALIX form condensates *in vitro.*

(a) Purification of recombinant ALIX and CaLB1 used for the in vitro analysis shown in Fig. 7b-h and in (c). Recombinant CaLB1 and ALIX were isolated from E. coli and resolved on an SDS-PAGE and protein bands were visualized by staining with CBB. Source data are provided as a Source Data file.

(b) Purification of recombinant CaLB1(C2) used for the in vitro analysis shown in Fig. 7b-d. Recombinant CaLB1(C2) were isolated from E. coli and resolved on an SDS-PAGE and protein bands were visualized by staining with CBB. Source data are provided as a Source Data file.

(c) Fluorescent recovery after photobleaching (FRAP) experiment of ALIX in condensates. ALIX was labelled with Alexa Fluor 488 and incubated in the presence of 5% PEG for 30 minutes. Photobleaching was performed with 100% laser power and the recovery of fluorescence was monitored by imaging 1 frame every 2 seconds. Arrowhead, bleached condensate. s: seconds. Data were normalized using the full-scale normalization method with the recovery curve starting from zero values. 20 condensates were analyzed. The line shows the mean value and error bands indicate the standard deviation. On the fit mean data (R Square: 0.99), the $\tau 1/2$ value (half time of recovery) and the proportion of the mobile fraction were calculated. s: seconds. Source data are provided as a Source Data file.

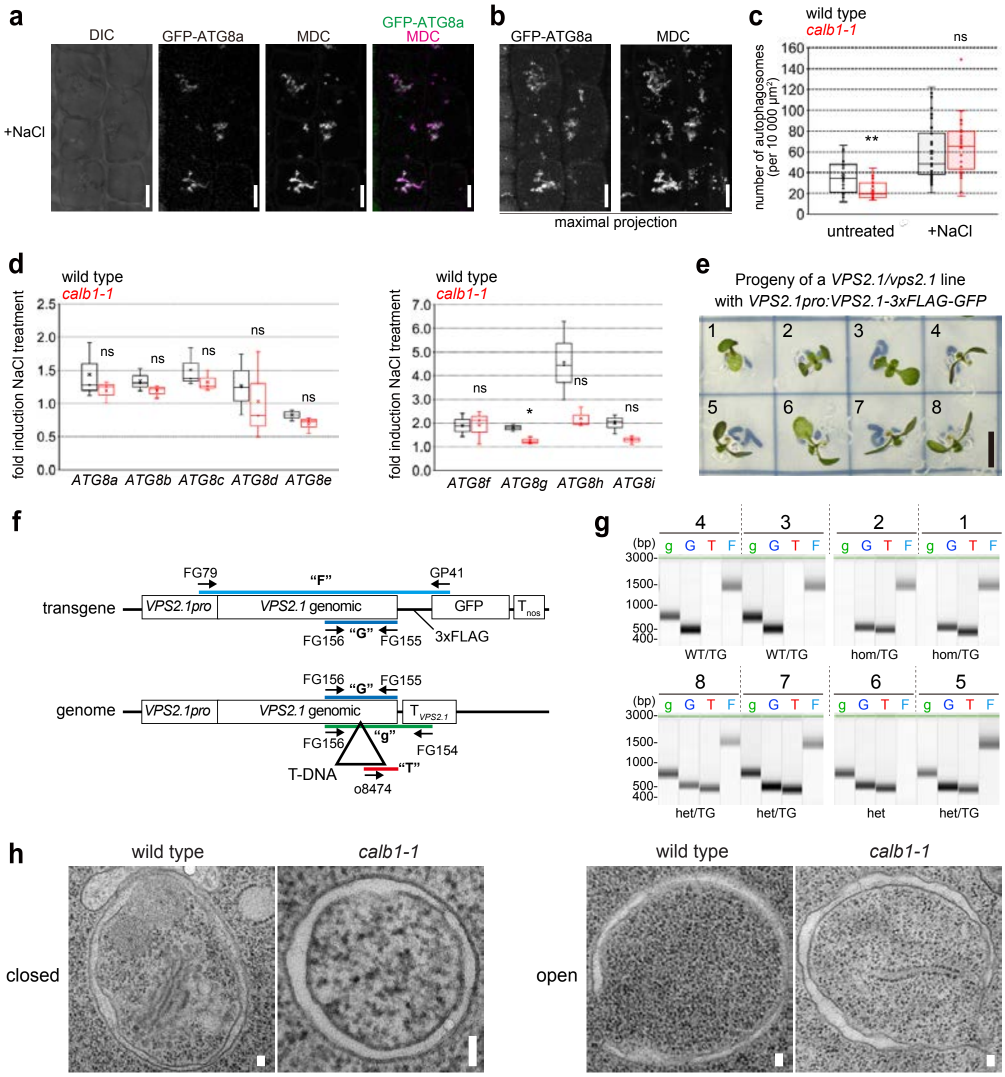

Supplementary Figure 8: *calb1-1* shows defects in autopahgy.

(a)(b) *UBQ10pro:GFP-ATG8a* expressing seedlings were treated with 100 mM NaCl for 2 days and with 100 µM E64d for 5 hours. Seedlings were subsequently stained with 50 µM MDC and root epidermis cells were imaged. Single slice images (a) and maximal projection images (b) are shown. is shown. For this experiment 4 different roots were analyzed with a confocal microscope. Scale bars: 10 µm.

(c) The number of autophagosomes as shown in Fig. 8d was normalized to 10 000 µm2. 18 and 17 seedlings were analyzed in the wild type and calb1-1 (untreated), respectively, and 35 seedlings each for wild type and *calb1-1* (+NaCl). Wild type/*calb1-1* (untreated) p=0.00827 (**: 0.001<p<0.01), wild type/*calb1-1* (+NaCl) p=0.437 (ns: not significant, p>0.5). The experiment was conducted three times.

(d) Expression of *ATG8*s in wild type and *calb1-1* upon NaCl treatment normalized against *ACTIN8*. Box plots show the results of three experiments each with technical quadruplicates. Wild type/*calb1-1*, for *ATG8a* p= 0.428 (ns: not significant, p>0.05), *ATG8b* p= 0.258 (ns, p>0.05), *ATG8c* p= 0.410 (ns, p>0.05), *ATG8d* p= 0.636 (ns, p>0.05), *ATG8e* p= 0.197 (ns, p>0.5), *ATG8f* p= 0.998 (ns, p>0.05), *ATG8g* p= 0.0116 (*, 0.01<p<0.05), *ATG8h* p= 0.0838 (ns, p>0.05), *ATG8i* p= 0.159 (ns, p>0.05).

(e) A photograph of progenies from a VPS2.1/vps2.1 heterozygous plant containing a hemizygous *VPS2.1pro:VPS2.1-GFP* transgene. Scale bar: 5 mm.

(f) Scheme of primer positions for the genotyping in the *VPS2.1pro:VPS2.1-3xFLAG-GFP* construct and in the genomic region containing *VPS2.1*. Tnos: nos terminator. The colored lines indicate the PCR product as follows: blue: "g", green: "G", red: "T", and light blue: "F".

(g) Genotyping of the seedlings shown in (e). g: genomic fragment flanking the T-DNA insertion site specific to genomic *VPS2.1*, G: genomic fragment from both the transgene and the genomic *VPS2.1*, T: T-DNA, F: Fluorophore (GFP)-containing fragment specific to the transgene. Note that the absence of the genomic fragment "g" and presence of the T-DNA "T" indicates the homozygosity for the T-DNA insertion in *VPS2.1*, and the "F" PCR product the presence of the GFP-fusion construct.

(h) Representative electron micrographs of the autophagosomes from wild type and *calb1-1* analyzed in Fig.8i. 5-day-old seedlings were transferred to liquid 1/2 MS containing 150 mM NaCl for 2 hours prior to cryofixation. 50 nm ultra-thin slices were prepared from four seedling roots. Scale bars: 100 nm.

(c, d) Box plot: center line, median; box limits, first and third quartiles; whiskers, 1.5x interquartile range. Two-tailed t-test, no equal variance.

(c, d, g) Source data are provided as a Source Data file.

Supplementary References

1. Nakagawa, T. *et al.* Development of series of gateway binary vectors, pGWBs, for realizing efficient construction of fusion genes for plant transformation. *J Biosci Bioeng* **104**, 34-41, 10.1263/jbb.104.34, (2007).
2. Shimada, T.L., Shimada, T. & Hara-Nishimura, I. A rapid and non-destructive screenable marker, FAST, for identifying transformed seeds of Arabidopsis thaliana. *Plant J* **61**, 519-528, 10.1111/j.1365-313X.2009.04060.x, (2010).
3. Wang, Z.P. *et al.* Egg cell-specific promoter-controlled CRISPR/Cas9 efficiently generates homozygous mutants for multiple target genes in Arabidopsis in a single generation. *Genome Biol* **16**, 144, 10.1186/s13059-015-0715-0, (2015).
4. Binder, A. *et al.* A modular plasmid assembly kit for multigene expression, gene silencing and silencing rescue in plants. *PLoS ONE* **9**, e88218, 10.1371/journal.pone.0088218, (2014).
5. Grefen, C. *et al.* A ubiquitin-10 promoter-based vector set for fluorescent protein tagging facilitates temporal stability and native protein distribution in transient and stable expression studies. *Plant J* **64**, 355-365, 10.1111/j.1365-313X.2010.04322.x, (2010).
6. Kalinowska, K. *et al.* Arabidopsis ALIX is required for the endosomal localization of the deubiquitinating enzyme AMSH3. *Proc Natl Acad Sci U S A* **112**, E5543-5551, 10.1073/pnas.1510516112, (2015).
7. Vogel, K. *et al.* Lipid-mediated activation of plasma membrane-localized deubiquitylating enzymes modulate endosomal trafficking. *Nat Commun* **13**, 6897, 10.1038/s41467-022-34637-3, (2022).
