# Supplementary material for: Arabidopsis CaLB1 undergoes phase separation with the ESCRT protein ALIX and modulates autophagosome maturation
Source: Nat Commun. 2024 Jun 19;15:5188. doi: 10.1038/s41467-024-49485-6 (PMC11187125; doi:10.1038/s41467-024-49485-6)
Supplement: Supplementary file 3 — Description of additional supplementary files document [file 41467_2024_49485_MOESM3_ESM.pdf]

<u>Description of Additional Supplementary Files:</u>

Supplementary Movie 1: CaLB1 puncta on ATG8a-marked autophagosomes.
Time lapse images of 5-day-old seedlings expressing CaLB1pro:CaLB1-GFP together with UBQ10pro:mRFP-ATG8a treated for 2 hours with 150 mM NaCl before imaging. Confocal images were taken with a frame interval of 1.7 seconds.

Supplementary Movie 2: Dissociation of CaLB1 puncta from autophagic membranes.
Time lapse images of 5-day-old seedlings expressing CaLB1pro:CaLB1-GFP together with UBQ10pro:mRFP-ATG8a treated for 2 hours with 150 mM NaCl before imaging. Time series was taken with a frame interval of 2.21 seconds.

Supplementary Movie 3: Appearance of CaLB1 on autophagic membranes.
Time lapse images of 5-day-old seedlings expressing CaLB1pro:CaLB1-GFP together with UBQ10pro:mRFP-ATG8a treated for 2 hours with 150 mM NaCl before imaging. Time series was taken with a frame interval of 1.7 seconds.
