# Supplementary material for: Arabidopsis CaLB1 undergoes phase separation with the ESCRT protein ALIX and modulates autophagosome maturation
Source: Nat Commun. 2024 Jun 19;15:5188. doi: 10.1038/s41467-024-49485-6 (PMC11187125; doi:10.1038/s41467-024-49485-6)
Supplement: Supplementary file 7 — Reporting Summary [file 41467_2024_49485_MOESM7_ESM.pdf]

Corresponding author(s): Erika Isono
Last updated by author(s): May 20, 2024

Reporting Summary

Nature Portfolio wishes to improve the reproducibility of the work that we publish. This form provides structure for consistency and transparency in reporting. For further information on Nature Portfolio policies, see our Editorial Policies and the Editorial Policy Checklist.

Statistics

For all statistical analyses, confirm that the following items are present in the figure legend, table legend, main text, or Methods section.

n/a	Confirmed	
☐	☒	The exact sample size (n) for each experimental group/condition, given as a discrete number and unit of measurement
☐	☒	A statement on whether measurements were taken from distinct samples or whether the same sample was measured repeatedly
☐	☒	The statistical test(s) used AND whether they are one- or two-sided<br>*Only common tests should be described solely by name; describe more complex techniques in the Methods section.*
☒	☐	A description of all covariates tested
☒	☐	A description of any assumptions or corrections, such as tests of normality and adjustment for multiple comparisons
☐	☒	A full description of the statistical parameters including central tendency (e.g. means) or other basic estimates (e.g. regression coefficient) AND variation (e.g. standard deviation) or associated estimates of uncertainty (e.g. confidence intervals)
☐	☒	For null hypothesis testing, the test statistic (e.g. F, t, r) with confidence intervals, effect sizes, degrees of freedom and P value noted<br>*Give P values as exact values whenever suitable.*
☒	☐	For Bayesian analysis, information on the choice of priors and Markov chain Monte Carlo settings
☒	☐	For hierarchical and complex designs, identification of the appropriate level for tests and full reporting of outcomes
☒	☐	Estimates of effect sizes (e.g. Cohen's d, Pearson's r), indicating how they were calculated

Our web collection on statistics for biologists contains articles on many of the points above.

Software and code

Policy information about availability of computer code

Data collection	Amersham Imager 600 1.2.0 (Cytiva), Zen Black 2.3 (Zeiss), Bio-Rad CFX Maestro 1.1, Tecan Magellan Pro 7.3 (Tecan), MO.Control x86 1.5.1. (NanoTemper), MO.Control v2.5.4 (NanoTemper), TEM Center for Jeol 2100 Plus (Jeol), Xepr v 2.7 (Bruker)
Data analysis	Excel 2016 (Microsoft), RStudio Team (2020). RStudio: Integrated Development for R. RStudio, PBC, Boston, MA URL [http://www.rstudio.com/], ImageJ 2.14.0/1.54f [https://imagej.net/ij/], Amersham Imager analysis software 1.0 (Cytiva), Alphafold [https://alphafold.ebi.ac.uk], UCFS ChimeraX-1.6.1 [https://www.rbvi.ucsf.edu/chimerax], Photoshop 2020 (Adobe), Illustrator 2020 (Adobe), MO.AffinityAnalysis x86 2.2.4 (NanoTemper), MO.Control v2.5.4 (NanoTemper), Opus Package (Bruker Optics), ZEN Black 2.3 (Zeiss), IUPred3 [https://iupred3.elte.hu], easyFRAP [https://easyfrap.vmnet.upatras.gr], Geneious Prime 2023.2.1 [https://www.geneious.com], MatLab R2022a [https://www.mathworks.com/products/new_products/release2022a.html], EasySpin 5.2.25 [https://easyspin.org], ComparativeDeerAnalyzer within DeerAnalysis 2022, Phytozome web [https://phytozome-next.jgi.doe.gov], TAIR Blast [https://www.arabidopsis.org/Blast/], SGD [https://www.yeastgenome.org], GenBank [https://www.ncbi.nlm.nih.gov/genbank/], Uniprot [https://www.uniprot.org], Alphafold II [https://alphafold.ebi.ac.uk], iLIR autophagy database [https://ilir.warwick.ac.uk], IUPred [https://iupred3.elte.hu] and ANCHOR [http://anchor.elte.hu]

For manuscripts utilizing custom algorithms or software that are central to the research but not yet described in published literature, software must be made available to editors and reviewers. We strongly encourage code deposition in a community repository (e.g. GitHub). See the Nature Portfolio guidelines for submitting code & software for further information.

Data

Policy information about availability of data

All manuscripts must include a data availability statement. This statement should provide the following information, where applicable:

- Accession codes, unique identifiers, or web links for publicly available datasets
- A description of any restrictions on data availability
- For clinical datasets or third party data, please ensure that the statement adheres to our policy

The data that support the findings of this study are available from the corresponding author upon request. Source files for immunoblots, protein gels, and graphs are provided with this paper in the Source Data file. In this current studies we used the databases Uniprot [https://www.uniprot.org], TAIR [https://www.arabidopsis.org/index.jsp], Phytozome 91 (https://phytozome-next.jgi.doe.gov), Saccharomyces Genome Database [https://www.yeastgenome.org], the UCSC genome browser [https://genome.ucsc.edu/index.html] or GenBank [https://www.ncbi.nlm.nih.gov/genbank/] and AlphaFold II [https://alphafold.ebi.ac.uk].

Research involving human participants, their data, or biological material

Policy information about studies with human participants or human data. See also policy information about sex, gender (identity/presentation), and sexual orientation and race, ethnicity and racism.

Reporting on sex and gender	*Use the terms sex (biological attribute) and gender (shaped by social and cultural circumstances) carefully in order to avoid confusing both terms. Indicate if findings apply to only one sex or gender; describe whether sex and gender were considered in study design; whether sex and/or gender was determined based on self-reporting or assigned and methods used. Provide in the source data disaggregated sex and gender data, where this information has been collected, and if consent has been obtained for sharing of individual-level data; provide overall numbers in this Reporting Summary. Please state if this information has not been collected. Report sex- and gender-based analyses where performed, justify reasons for lack of sex- and gender-based analysis.*
Reporting on race, ethnicity, or other socially relevant groupings	*Please specify the socially constructed or socially relevant categorization variable(s) used in your manuscript and explain why they were used. Please note that such variables should not be used as proxies for other socially constructed/relevant variables (for example, race or ethnicity should not be used as a proxy for socioeconomic status). Provide clear definitions of the relevant terms used, how they were provided (by the participants/respondents, the researchers, or third parties), and the method(s) used to classify people into the different categories (e.g. self-report, census or administrative data, social media data, etc.) Please provide details about how you controlled for confounding variables in your analyses.*
Population characteristics	*Describe the covariate-relevant population characteristics of the human research participants (e.g. age, genotypic information, past and current diagnosis and treatment categories). If you filled out the behavioural & social sciences study design questions and have nothing to add here, write "See above."*
Recruitment	*Describe how participants were recruited. Outline any potential self-selection bias or other biases that may be present and how these are likely to impact results.*
Ethics oversight	*Identify the organization(s) that approved the study protocol.*

Note that full information on the approval of the study protocol must also be provided in the manuscript.

Field-specific reporting

Please select the one below that is the best fit for your research. If you are not sure, read the appropriate sections before making your selection.

☒ Life sciences ☐ Behavioural & social sciences ☐ Ecological, evolutionary & environmental sciences

For a reference copy of the document with all sections, see nature.com/documents/nr-reporting-summary-flat.pdf

Life sciences study design

All studies must disclose on these points even when the disclosure is negative.

Sample size	Sample size calculation was not performed prior to the experiments. The sample size was determined based on the experience of similar assays performed earlier. The experiments were reproduced as indicated in the manuscript
Data exclusions	Root length analyses: seeds that were not germinated or seedlings that show an arrested growth soon after germination were excluded from all replicates. FRAP experiments: frames taken after 200 seconds were not analyzed to avoid effects of photobleaching occurring during imaging. ATR-IR Spectroscopy: data over 90 minutes were not included in the analysis.
Replication	All controls and treated samples were handled together in a single experiment. Yeast two-hybrid experiments were conducted using three indipendent transformants. All in vitro pull down assays and in vitro lipid overlay assays were conducted at least twice with consistent results. Analysis on the colocalization between CaLB and ALIX was conducted three times. The ATR-IR spectroscopy experiment was conducted independently three times. The MST analyses include at least two measurements with consistent results. The qRT-PCR analysis were performed three times. Immunoblot on the induction of ALIX, CaLB and ATG8a was performed three times. Root length analyses were

performed at least twice with consistent results. Confocal study on salt-treated CaLB-GFP, GFP-ATG8 and GFP-ALIX lines was conducted 6 times with consistent results. Analysis on the colocalization between CaLB and ALIX upon salt was conducted three times. Colocalization analyses between CaLB and either ATG8a or ATG8i were conducted three times. Colocalization analysis between CaLB and ATG8e was conducted on two different independent lines. Time-lapse images were collected over three different experiments with consistent results. Analysis on the colocalization between CaLB(F64A)-GFP and mRFP-ATG8i was conducted on seedlings from three independent lines. Analysis on the colocalization between ALIX and ATG8 were conducted on 11 seedlings. Autophagosome fractionation upon salt treatment was performed twice and immunoblots showed consistent results. 1,6-hexanediol treatments were conducted at least twice with consistent results. Immunoblot on the protein extract from protoplast was repeated twice with consistent results. Over 100 protoplasts were analyzed [n=120 (CRISPR), n=111(mutCRISPR)]. Experiment on protoplasts isolated from wild type or calb1-1 roots was replicated four times. Condensates experiments were reproduced at least twice with consistent results. The confocal analyses on E64d treated seedlings was conducted twice with reproducible results. The E64d experiments on GFP-ATG8a and atg10-1 were conducted on at least 4 different seedlings each. The experiment that show the number of autophagosomes in wild type and mutant background was repeated three times. The GFP cleavage assays were reproduced at least three times. The immunoblot relative to the protease protection assay was reproduced four times. The colocalization analyses between VPS2.1 and GFP-ATG8a was conducted on 18 independent lines (wt background) and on 14 independent lines (calb1-1 background). The TEM analysis on autophagosome closure in wt and calb1-1 roots was conducted from 4 independent roots each embedded in four different resin blocks. The treatment of seedlings with WM, BFA, and E64d were conducted at least twice showing consistent results. Chlorophyll assay were conducted three times. La(III)Cl experiment was conducted three times the average value for each experiment was calculated and shown. Hexanediol recovery experiment was repeated three times. Colocalization between ATG8i and either CaLB or ALIX (control treatment -NaCl) was conducted three times.

Randomization: No randomization was applicable since there was no organization in experimental groups.

Blinding: Blinding was not performed since there was no organization in experimental groups.

Reporting for specific materials, systems and methods

We require information from authors about some types of materials, experimental systems and methods used in many studies. Here, indicate whether each material, system or method listed is relevant to your study. If you are not sure if a list item applies to your research, read the appropriate section before selecting a response.

Materials & experimental systems

n/a	Involved in the study
☐	☒ Antibodies
☒	☐ Eukaryotic cell lines
☒	☐ Palaeontology and archaeology
☒	☐ Animals and other organisms
☒	☐ Clinical data
☒	☐ Dual use research of concern
☐	☒ Plants

Methods

n/a	Involved in the study
☒	☐ ChIP-seq
☒	☐ Flow cytometry
☒	☐ MRI-based neuroimaging

Antibodies

Antibodies used

Primary antibodies:
anti-ALIX - described in our previous publication
anti-H+-ATPase (5000× diluted, Agrisera, AS07260)
anti-FLAG (M2) (1000× diluted, Sigma Aldrich, F1804)
anti-GBD (3000× diluted, Santa Cruz, sc-510)
anti-GFP (1000× diluted, 3H9,Chromotek, 3H9-100)
anti-GST (1000× diluted, Eurogentec) - described in our previous publication
anti-His (1000× diluted, Thermo Fisher, P-21315)
anti-HA(3F10) (1000× diluted, Roche, 11867423001)
anti-MBP (10,000× diluted, NEB, E8032S)
anti-RFP (1000× diluted, Cromotek, 6G6)
anti-UGPase (3000× diluted, Agrisera, AS05086)
anti-NBR1 (2000× diluted, Agrisera, AS142805A)
anti-H3 (5000× diluted, Agrisera, AS10710)
anti-ACTIN (50× diluted, JLA20, Sigma Aldrich)
anti-CDC2 (5000× diluted, Santa Cruz, sc-166885)
anti-GFP antibody (1000× diluted, Abcam, ab13970)

Secondary antibodies:
anti-rat-HRP (80,000×diluted, Roche, A9037)
anti-mouse-HRP (80,000× diluted, Sigma Aldrich, A9044)
anti-rabbit-HRP (80,000× diluted, Sigma Aldrich, A0545)
anti-chicken antibody conjugated with 12 nm gold particles (10× diluted, Dianova, 703-205-155)
anti-rabbit-Alkaline Phosphatase (AP) (30000× diluted, Sigma Aldrich, A3812)
anti-rat-AP (30000× diluted, Sigma Aldrich, A6066)

Validation

Validation statements (URL) for primary antibodies:

Validation

Primary antibodies were verified by comparison with the molecular weight marker and comparison with appropriate negative controls.

anti-H+-ATPase (rabbit) polyclonal, Agrisera, AS07260 - Datasheet of the product is available under the following link: [https ://www. agrisera.com/ cgi-bin/ibutik/SkapaFaktura.pl?SkrivPDF=J&artnr=AS07%20260&Friendly=hatpase-plasma-membrane-hatpase&skrivpdf=j&Friendly_ Grupp=&funk=visa_artikel&Sprak= EN&artgrp=43]

antiFLAG (M2), monoclonal, Sigma-Aldrich MABT219 - Validation statement of the product is available under the following link: [https://www.sigmaaldrich.com/specification-sheets/469/360/F1804-5MG__________SIGMA_____.pdf]

anti-GBD, Santa Cruz, sc-510, mouse monoclonal - Datasheet of the product is available under the following link: [https://datasheets.scbt.com/sc-510.pdf])

anti-GFP (rat) [3H9], monoclonal, Chromotek, 3H9-100 - Datasheet of the product is available under the following link: [https://www.chromotek.com/fileadmin/content/Images/ Antibodies lgGs/GFP_3H9/029762_ validation_report_ Chromotek.pdf])

anti-His, Thermo Fisher, P-21315, mouse monoclonal - Validation statement of the product is available under the following link: [https://www.thermofisher.com/antibody/product/Penta-His-Tag-Antibody-Monoclonal/P-21315]

anti-HA(3F10), Roche, 11867423001, rat monoclonal - Validation statement of the product is available under the following link: [https://www.sigmaaldrich.com/DE/en/product/roche/roahaha]

anti-MBP, NEB, E8032S, monoclonal murine antibody - Specification sheet of this product available at the following link: [https://international.neb.com/-/media/catalog/specifications/e8032s_l_v1.pdf?rev=d1019486ebb743edacb255bb97dd191d&hash=23219A630ACA97DDB5D64D32D073D071]

anti-RFP, Cromotek, 6G6, mouse monoclonal - Datasheet of the product is available under the following link: [https://www.ptglab.com/products/pictures/pdf/6g6_Datasheet_RFP%20antibody%20[6G6].PDF]

anti-UGPase (rabbit), polyclonal, Agrisera, AS05086 - Datasheet of the product is available under the following link: [https ://www. agrisera.com/ cgi-bin/ibutik/SkapaFaktura.pl?SkrivPDF=J&artn=AS05%20086&skrivpdf=j&Friendly=ugpase-udp-glucosepyrophosphorylase-marker -of-cytoplasm&funk=visa_artikel&Sprak= EN&artgrp=9]

anti-NBR1 , Agrisera, AS142805A - Datasheet of the product is available under the following link: [https://www.agrisera.com/cgi-bin/ibutik/SkapaFaktura.pl?SkrivPDF=J&Sprak=EN&artgrp=116&Friendly_Grupp=&skrivpdf=j&artnr=AS14%202805&Friendly=nbr1&funk=visa_artikel]

anti-H3 Agrisera, AS10710 - Datasheet of the product is available under the following link: [https://www.agrisera.com/cgi-bin/ibutik/SkapaFaktura.pl?SkrivPDF=J&artnr=AS10%20710&Friendly=h3-histone-h3&funk=visa_artikel&Sprak=EN&artgrp=98&Friendly_Grupp=&skrivpdf=j]

anti-ACTIN JLA20, Sigma Aldrich - Datasheet of the product is available under the following link: [https://www.sigmaaldrich.com/DE/de/product/mm/mabt219?utm_source=google&utm_medium=cpc&utm_campaign=12478270955&utm_content=119588671918&gclid=EAIaIQobChMI6czZsY_8gwMVPJaDBx3YCwUWEAAYASAAEgJQMfD_BwE&icid=sharepdp-clipboard-copy-productdetailpage]

anti-CDC2 Santa Cruz, sc-166885 - Datasheet of the product is available under the following link: [https://datasheets.scbt.com/sc-166885.pdf]

anti-GFP antibody, Abcam, ab13970 - Datasheet of the product is available under the following link: [https://www.abcam.com/en-de/products/primary-antibodies/gfp-antibody-ab13970]

anti-rat HRP, Roche, A9037 - Datasheet of the product is available under the following link: [https://www.sigmaaldrich.com/DE/en/product/sigma/a9037?utm_source=google&utm_medium=cpc&utm_campaign=12410876063&utm_content=120911518523&gclid=EAIaIQobChMInMDU6pGUhQMVK2hBAh3d0QbFEAAYASAAEgI3b_D_BwE]

anti-mouse-HRP, Sigma Aldrich, A9044 - Datasheet of the product is available under the following link: [https://www.sigmaaldrich.com/DE/en/product/sigma/a9044?utm_source=google&utm_medium=cpc&utm_campaign=12410876063&utm_content=120911518723&gclid=EAIaIQobChMIrK6Ur5KUhQMVnZGDBx1yfAkkEAAYASAAEgIkNvD_BwE#product-documentation]

anti-rabbit-HRP , Sigma Aldrich, A0545 - Datasheet of the product is available under the following link: [https://www.sigmaaldrich.com/DE/en/product/sigma/a0545?utm_source=google&utm_medium=cpc&utm_campaign=12478270955&utm_content=119588671918&gclid=EAIaIQobChMI2Njo2pKUhQMV-6KDBx1tuQ-lEAAYAyAAEgIChvD_BwE]

anti-chicken antibody conjugated with 12 nm gold particles, Dianova, 703-205-155 - Datasheet of the product is available under the following link: [https://www.dianova.com/downloads/Jackson/703-205-155.pdf]

anti-rabbit-AP, Sigma Aldrich, A3812 - Datasheet of the product is available under the following link: [https://www.sigmaaldrich.com/DE/en/product/sigma/a3812]

anti-rat-AP, Sigma Aldrich, A6066 - Datasheet of the product is available under the following link:

[https://www.sigmaaldrich.com/DE/en/product/sigma/a6066]

Dual use research of concern

Policy information about dual use research of concern

Hazards

Could the accidental, deliberate or reckless misuse of agents or technologies generated in the work, or the application of information presented in the manuscript, pose a threat to:

No	Yes	
☒	☐	Public health
☒	☐	National security
☒	☐	Crops and/or livestock
☒	☐	Ecosystems
☒	☐	Any other significant area

Experiments of concern

Does the work involve any of these experiments of concern:

No	Yes	
☒	☐	Demonstrate how to render a vaccine ineffective
☒	☐	Confer resistance to therapeutically useful antibiotics or antiviral agents
☒	☐	Enhance the virulence of a pathogen or render a nonpathogen virulent
☒	☐	Increase transmissibility of a pathogen
☒	☐	Alter the host range of a pathogen
☒	☐	Enable evasion of diagnostic/detection modalities
☒	☐	Enable the weaponization of a biological agent or toxin
☒	☐	Any other potentially harmful combination of experiments and agents
